# First molecular evidence of *Leishmania* parasites in sand flies (Diptera: Phlebotominae) from Slovenia

**DOI:** 10.1186/s13071-025-07006-4

**Published:** 2025-08-22

**Authors:** Gioia Bongiorno, Katja Adam, Ilaria Bernardini, Claudia Mangiapelo, Eleonora Fiorentino, Trentina Di Muccio, Vladimir Ivović

**Affiliations:** 1 Department of Infectious Diseases, Vector-Borne Diseases Unit, Istituto Superiore di Sanità, Rome, Italy; 2 Faculty of Mathematics, Natural Sciences and Information Technologies, University of Primorska, Koper, Slovenia

**Keywords:** Sand flies, *Leishmania*, Slovenia, Vector surveillance, Seasonality

## Abstract

**Background:**

Sand flies (Diptera: Phlebotominae) are vectors of *Leishmania* spp., protozoan parasites that cause leishmaniasis, a zoonosis endemic in the Mediterranean region. Although Slovenia is not considered endemic, its proximity to affected areas and the presence of competent vectors underscore the importance of entomological surveillance. As part of the CLIMOS project, we investigated sand fly species composition, seasonal abundance, and the presence of *Leishmania* parasites at two sites in southwestern Slovenia: Cetore and Velike Žablje.

**Methods:**

From May to October 2023, adult sand flies were collected using Centers for Disease Control and Prevention (CDC) miniature light traps. Specimens were morphologically identified and screened for *Leishmania* spp. DNA using quantitative polymerase chain reaction (PCR) targeted kinetoplast DNA, followed by species confirmation with ITS-1 nested PCR and restriction fragment length polymorphism (RFLP) analysis.

**Results:**

A total of 274 sand flies were collected, predominantly *Phlebotomus neglectus*, *P. perniciosus*, and *P. mascittii*. Abundance peaked in July in Cetore and August in Velike Žablje, likely reflecting local climatic differences. Among 25 pools of female sand flies analyzed, *Leishmania* DNA was detected in three pools (12.0%), specifically in *P. neglectus* and *P. mascittii*. The cycle threshold values (Ct 35–37) indicated low parasite DNA loads. ITS-1 amplification was unsuccessful, likely due to low DNA concentration.

**Conclusions:**

This study provides the first molecular evidence of *Leishmania* DNA in sand flies from Slovenia. The detection of *Leishmania* in *P.  mascittii*, a species with uncertain vector competence, is particularly noteworthy and warrants further investigation. Although species identification of the parasite was not possible, the findings suggest potential for autochthonous transmission and highlight the need for continued surveillance and research in the region.

**Graphical Abstract:**

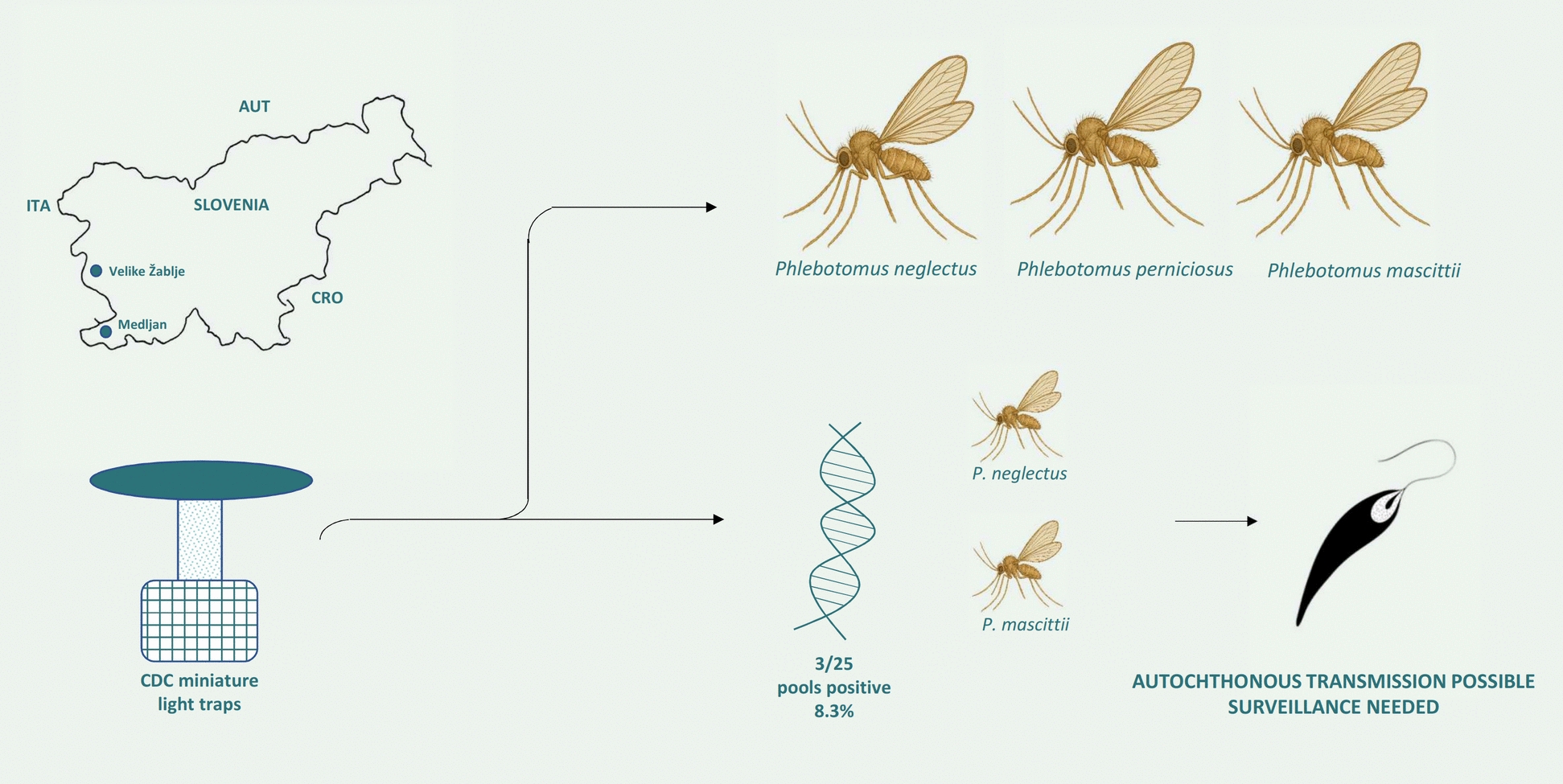

**Supplementary Information:**

The online version contains supplementary material available at 10.1186/s13071-025-07006-4.

## Background

Sand flies (Diptera: Psychodidae: Phlebotominae) are arthropods of great importance to public health and veterinary medicine worldwide, due to their role as vectors of various pathogens that affect both humans and animals. They are proven vectors of several *Leishmania* species, associated with various clinical forms of the disease [7, 17, 18] as well as several medically relevant phleboviruses, including Toscana virus, sand fly fever Sicilian virus, and sand fly fever Naples virus [11]. They are also involved in the transmission of *Bartonella bacilliformis*, the causative agent of bartonellosis [22].

Leishmaniasis is a vector-borne disease caused by protozoan parasites of the genus *Leishmania* and transmitted to humans and animals through the bite of infected female sand flies. In the Old World, the disease mainly manifests itself in two forms: visceral leishmaniasis (VL), which is associated with systemic impairment and can be fatal if untreated, and cutaneous leishmaniasis (CL), which is characterized by skin lesions [7].

The Mediterranean region is endemic for leishmaniasis, with several hotspots particularly concentrated around the Mediterranean basin and the Black Sea region [18, 25]. The endemic *Leishmania* species include *Leishmania donovani* sensu lato (s.l.) (a species complex of *L. donovani* sensu stricto (s.s.) and *L. infantum*), as well as *L. tropica* and *L. major* [24]. In humans, the most common clinical forms of leishmaniasis are VL, which is mainly associated with *L. infantum* and *L. donovani* s.l., and CL, which can be caused by any of the four species. In animals, *L. infantum* is the predominant pathogen, with the dog being the most susceptible host. In Italy, the disease is widespread and numerous cases are reported each year [38]. Spain also reports a considerable number of cases, especially in the central and southern regions [8]. In France, cases have been documented mainly in southeastern areas [29], while Greece [37] and Albania have reported sporadic cases on the mainland and in coastal regions [41]. In Croatia, cases of leishmaniasis have been reported in both humans and dogs, particularly on the Dalmatian coast [9, 33]. It should be noted that zoonotic cutaneous leishmaniasis (ZCL) on the southern coast of the Mediterranean region is predominantly caused by *Leishmania major*, which is mainly transmitted by *Phlebotomus* (*Phlebotomus*) *papatasi*. This association has been confirmed by several authors, including Ben Ismail et al. [6], who isolated *L. major* from *P. papatasi*, and Chelbi et al. [12], who demonstrated a spatial correlation between the distribution of *P. papatasi* and the incidence of ZCL in Tunisia.

Outside the Mediterranean region, cases of leishmaniasis have been reported in neighboring countries. In Bulgaria, *Leishmania* parasites have not been isolated from sand flies, although sporadic human cases are reported annually in the southern part of the country [34]. In Serbia, leishmaniasis has been known since after the Second World War [39]. Although there is serious evidence that Kosovo is an endemic region for VL and autochthonous transmission has been reported in recent years, there are few data on human and canine cases and comparative figures from neighboring countries suggest significant underreporting [40].

In Europe, *Leishmania infantum* is the predominant species responsible for both VL and CL, with domestic dogs serving as primary reservoir hosts [43]. The European Centre for Disease Prevention and Control (ECDC) has identified several countries with varying degrees of endemicity, highlighting the need for continued surveillance and research [13]. Understanding the distribution of sand flies and associated *Leishmania* parasites is crucial for implementing effective control measures and preventing the spread of leishmaniasis in the most affected countries.

In this study, we aimed to (i) monitor the seasonal activity and species composition of sand fly populations at two ecologically distinct sites in southwestern Slovenia and (ii) assess the presence of *Leishmania* parasites in local sand fly specimens using molecular diagnostic methods. In this way, we aimed to provide the first evidence for the spread of *Leishmania* in sand flies in Slovenia and to contribute to the understanding of vector-borne disease risk in the region.

## Methods

### Study area

**Figure 1:**
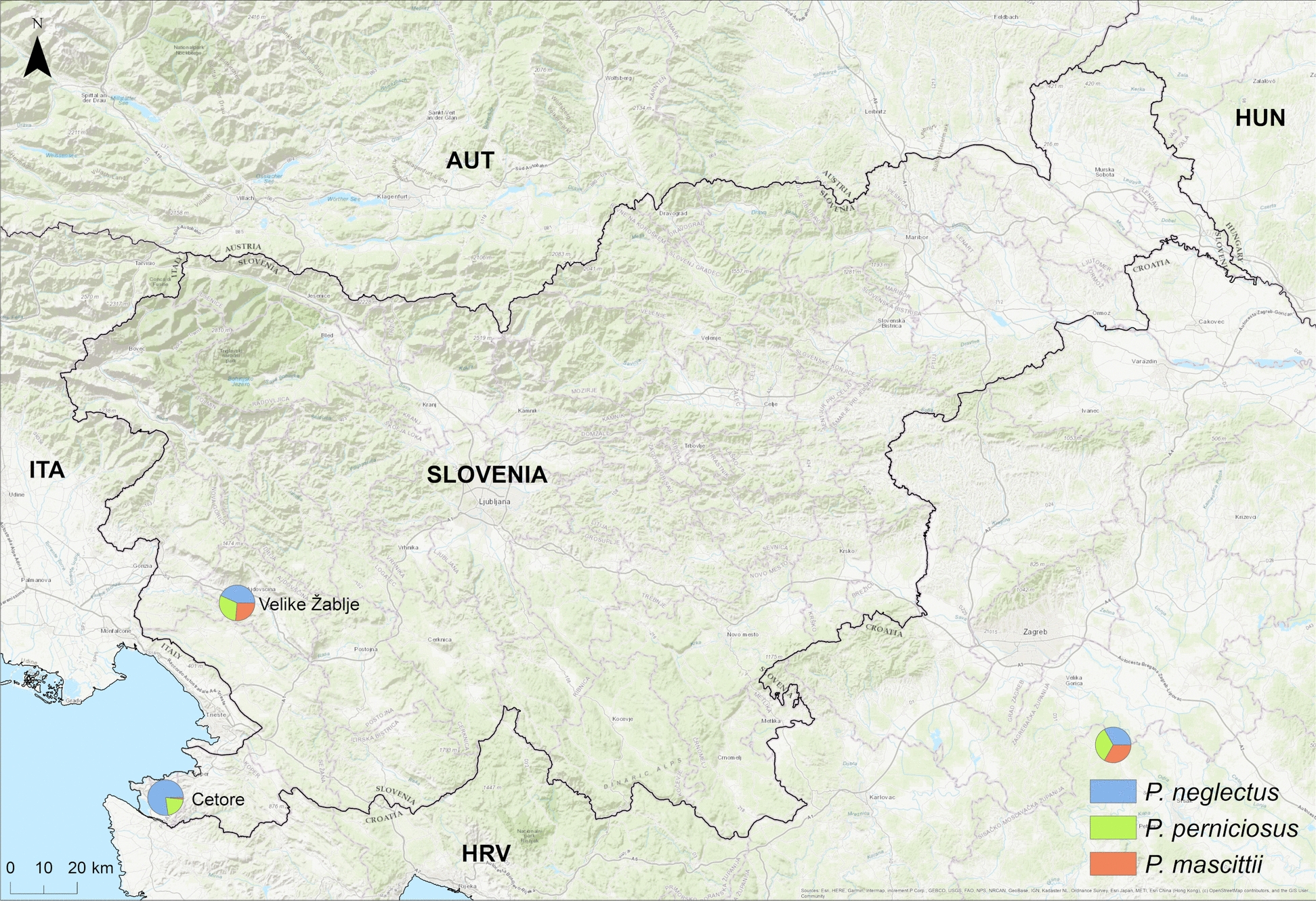
Map of Slovenian Istria and Karst including both study sites

As part of the CLIMOS project, the seasonal activity of sand flies was monitored at two sites in Slovenia: on the Medljan farm in Cetore (GPS 45.500396° N, 13.658786° E), which is located in the coastal part of Slovenia, and Velike Žablje (GPS 45.867859° N, 13.851472° E), which is located in the western part of the Slovenian Karst region (Fig. 1). These two sites were selected on the basis of the results of long-term, nationwide vector monitoring programmes, which identified them as hotspots with a high diversity and abundance of sand fly species. In addition, both sites harbor domestic animals and regular human activities that create favorable ecological conditions for the occurrence of vectors. At the Cetore site, the fauna included poultry, rabbits, donkeys, a dog, and humans. In Velike Žablje, in addition to human activities, domestic animals such as a dog, chickens, and rabbits were also present.

### Trapping methods

The fieldwork was carried out from 13 May to 10 October 2023, which corresponds to the seasonal sand fly activity in the study area on the coast and in the Karst region of Slovenia. Due to logistical and financial constraints caused by the distance between the laboratory and the sampling sites, the frequency of sampling varied from site to site. Two Centers for Disease Control and Prevention (CDC) miniature light traps (John W. Hock Company, Florida, USA) were set up twice a month at the Cetore site, which is about 3 km away from the laboratory, while sampling took place once a month at the Velike Žablje site, approximately 50 km away. In addition, CDC Gravid Traps for mosquitoes (John W. Hock Company, Florida, USA) were used as part of a broader entomological monitoring programme to monitor mosquito populations at the same sites. Although the CDC Miniature Light Traps were the primary method for collecting adult sand flies, they were supplemented by CDC Gravid Traps.

As sand flies are nocturnal, the traps were set around 7 p.m. and retrieved around 6 a.m. to maximize the chances of survival of the captured specimens. Since sand flies require sufficient moisture and an organic substrate to complete their life cycle, the traps were strategically placed near potential resting and breeding sites. At the Cetore site, one trap was placed near a chicken coop, directly under a fig tree, about 1 m above the ground. A second trap was placed 10 cm from a stone wall and 30 cm from the nearest rabbit cage at a height of 1.2 m, also under a fig tree branch. At the Velike Žablje site, a CDC trap was placed near the chicken coop at a height of about 1 m, and another one 50 cm from the rabbit cages at a height of 0.5 m. In addition, a CDC gravid trap was placed behind the farm building in the garden near a stone wall.

### Identification of sand flies based on morphology

The sand flies were identified on the basis of their morphological characteristics using identification keys [2, 23, 30].

As one of the aims of this study was to analyze the captured sand flies for the presence of *Leishmania* parasites and phleboviruses, all specimens were placed in a freezer immediately after returning from the field and stored at −80 °C until dissection. The dissections were performed on ice plates as soon as possible to preserve the viral RNA.

The head and last posterior abdominal segments of each specimen were carefully separated with fine entomological or insulin needles on a microscope slide. Specimens were then identified under a stereomicroscope using the above identification keys. The reproductive organs of each specimen were photographed, labeled, and stored in a database.

Female specimens in which the spermatheca was not visible were cleared by lactophenol solution for 24 h before identification under the microscope. The remaining body parts were pooled by location, date, species, and sex and stored at −80 °C for subsequent molecular analyses and detection of phlebovirus RNA.

### Molecular screening for Leishmania parasites

Total nucleic acid was extracted from the insect pools according to the instructions of the manufacturer of the Maxwell® 16 Cell DNA Purification Kit (Promega, Wisconsin, USA). *Leishmania* DNA was analyzed by quantitative polymerase chain reaction (qPCR) method on the basis of the amplification of a mini-circle sequence of kinetoplast DNA (kDNA) with primers and Taqman probe: 5ʹ-CTT-TTC-TGG-TCC-TCC-GGG-TAGG, 5ʹ-CCA-CCC-GGC-CCT-ATT-TTA-CAC-CAA and 5ʹ FAM-TTT-TCG-CAG-AAC-GCC-CCT-ACC-CGC-3ʹ TAMRA, respectively [27, 31]. Each 25 μl qPCR reaction mixture contained 5 μl DNA sample, 12.5 μl TaqMan Universal Master Mix 2X (Bio-Rad), and a final concentration of 0.5 μM of primers and 0.2 μM probe. DNA was amplified under the following conditions: initial step at 95 °C for 10 min, followed by 45 cycles of 15 s at 95 °C and 1 min at 60 °C. Amplification was performed in triplicate and included positive (*L. infantum*, MHOM/TN/80/IPT1 DNA) and negative (no DNA and DNA from reared *P. perniciosus*) controls. The qPCR cycle threshold (Ct), defined as the cycle at which near-logarithmic product amplification occurs, was used as a semi-quantitative measure of parasite DNA concentration. Samples with a Ct value of less than 40 were considered positive [30]. Molecular analyses performed on monospecific sand fly pools of up to 30 females allow estimation of the minimum infection rate (MIR), a conservative estimation method based on the assumption that only one infected sand fly occurs per positive pool, regardless of pool size [5].

Genotyping at the *Leishmania* species level was performed on the positive samples by ribosomal internal-transcribed spacer-1 (ITS-1) nested(n)-PCR, followed by restriction fragment length polymorphism (RFLP) analysis (ITS-1 n-PCR-RFLP) or sequencing [1]. All PCRs were performed in volumes of 50 µl PCR mix (GoTaq Green Master Mix, 2X Promega) with 2 µl DNA. The primers LITSR and L5.8 S were used to amplify a specific ITS-1 region in the ribosomal operon [32]. N-ITS-1 PCR was performed with 2 μl (or 5 μl in case of a negative result) of the previous ITS-1 PCR products, using the same primer combination and PCR conditions as for the first round of amplification. In case of negative results, a third round of amplification was performed under the same conditions. The PCRs were performed in the C1000 Thermal Cycler (BIORAD, CA, USA). A 2% agarose gel was used to check the size of the amplified product. A negative (no DNA, DNA from reared *P. perniciosus*) and a positive *Leishmania* DNA control were used for all amplifications.

## Results

### Sand fly species composition and abundance

**Figure 2:**
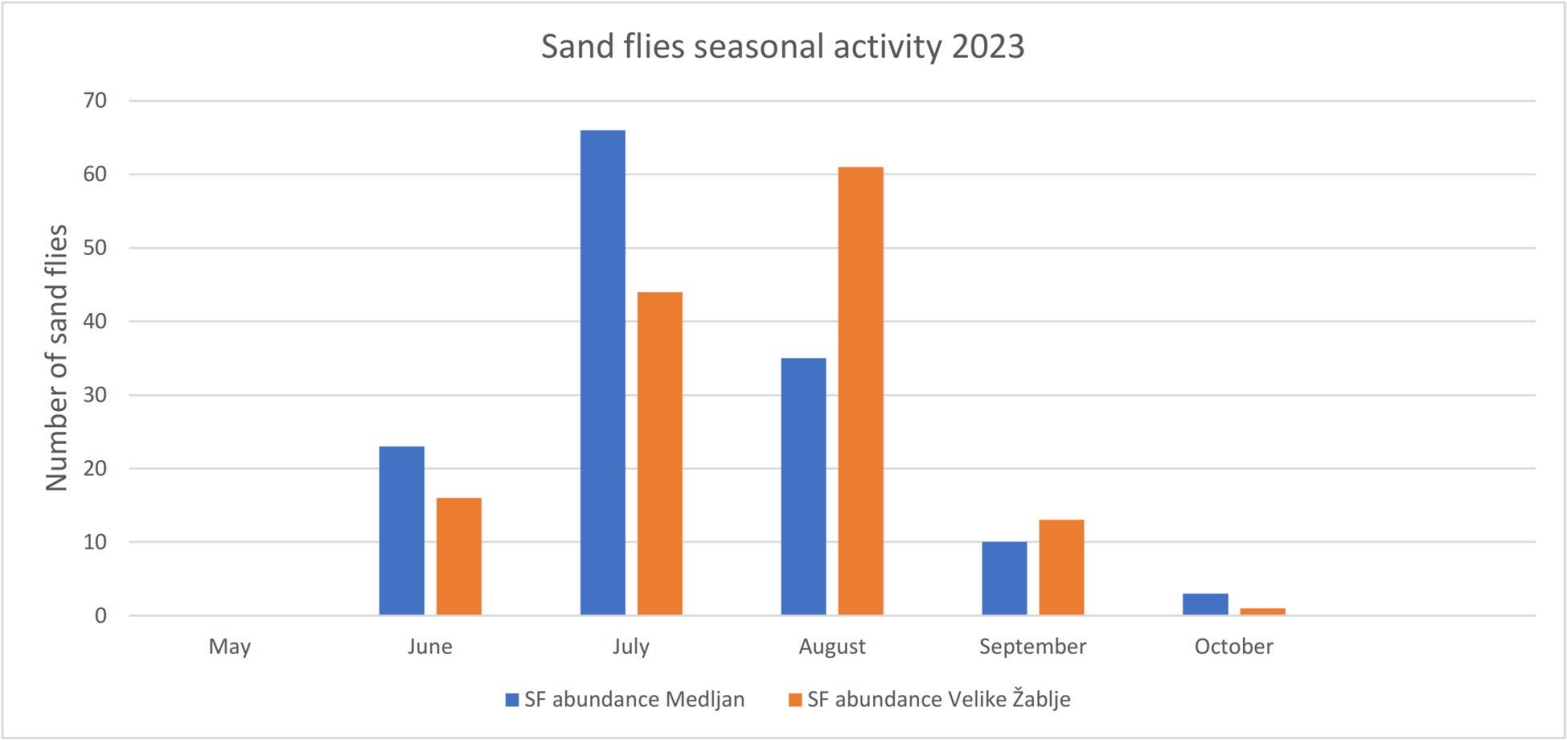
Seasonal abundance of sand flies at two sites, Cetore and Velike Žablje. In June, two female sand flies (one *P. neglectus* and one *P. mascittii*) tested positive in Cetore. In July, one female *P. mascittii* tested positive in Velike Žablje

In 2023, sand flies belonging to three species were collected at both sites: *Phlebotomus* (*Larroussius*) *neglectus*, *P.* (L.) *perniciosus*, and *P.* (*Transphlebotomus*) *mascittii*. A total of 133 specimens were caught in Cetore, with *P. neglectus* being the most abundant, followed by *P. perniciosus* and *P. mascittii*. Sand fly abundance peaked in July (*n* = 46) (Fig. 2). Males (*n* = 87) were more frequent than females (*n* = 44). *P. mascittii* was primarily detected in June and July, while *P. perniciosus* appeared from July to September (Table 1).

**Table 1:** Monthly distribution and sex ratio of *Phlebotomus neglectus*, *P. perniciosus*, and *P. mascittii* collected in Cetore and Velike Žablje (Slovenia), 2023. The total numbers are in bold.

CETORE	Date	*P*. *neglectus*				*P*. *perniciosus*		*P*. *mascitii*		Total	Females	Males
		f		m		f	m	f	m			
June	29.6	6		25		4	2	3		**40**	13	27
July	18.7	11		21		1	10	2	1	**46**	14	32
August	8.8	11		10		4	8	1		**34**	16	16
September	8.9			4			6			**10**	0	10
October	3.10			2		1				**3**	1	2
Abundance (%)				67.7			27.1		5.3			
VELIKE ŽABLJE												
May	13.5	0	0		0		0	0	0	**0**	0	0
June	15.6	3	1		1		0	11	0	**16**	15	1
July	17.7	14	8		5		7	9	1	**44**	28	16
August	17.8	10	18		8		12	12	1	**61**	30	31
September	22.9	1	3		2		5	2	0	**13**	5	8
October	10.10	0	1		1		0	0	0	**2**	1	1
Abundance (%)			43.4				30.1		26.5			

At Velike Žablje, 136 specimens were collected. *Phlebotomus neglectus* again dominated, followed by *P. mascittii* and *P. perniciosus*. The sex ratio was slightly male-biased (79 males versus 57 females). Peak abundance occurred in August (*n* = 61), with no captures in May and minimal abundance in October (Fig. 2). *P. mascittii* was most abundant between June and August, while *P. perniciosus* was present from June to September (Table 1).

### Molecular detection of Leishmania spp.

A total of 25 pools, comprising 36 female sand flies (25 from Cetore and 11 from Velike Žablje), were analyzed by qPCR. Leishmania DNA was detected in three pools (12.0%) (Table 2). Two positive pools were from Cetore (June), including one *P. neglectus* and one *P. mascittii* specimen, representing a positivity rate of 15.4% (2/13). One positive *P. mascittii* pool was identified in Velike Žablje (July), yielding a positivity rate of 11.1% (1/9). All three positive samples had Ct values between 35.0 and 37.0, indicating low parasite DNA loads (Additional files: Figs. A1–A3). Subsequent ITS-1 nPCRs were negative, consistent with limited template availability.

**Table 2:** *Leishmania* spp. positivity per collecting site, species, and month, based on *N* = number of analyzed pools (number of specimens in the pool). The pools in which Leishmania DNA was detected are in bold.

Site	Species	June		July	
		*N*	*Leishmania* spp. positivity	*N*	*Leishmania* spp. positivity
Cetore	*P. perniciosus*	3 (4)	0	1 (1)	0
	*P. mascittii*	**2 (3)**	**1 (1)**	0	0
	*P. neglectus*	**3 (6)**	**1 (1)**	3 (10)	0
	*Phlebotomus* spp.	0	0	1	0
Velike Žablije	*P. perniciosus*	0	0	1 (1)	0
	*P. mascittii*	8 (2)	0	**2 (4)**	**1 (1)**
	*P. neglectus*	0	0	1 (4)	0

## Discussion

To date, no autochthonous cases of human leishmaniasis have been reported in Slovenia; all confirmed cases have been imported from endemic Mediterranean regions [28]. However, sporadic cases of canine leishmaniasis have been documented over the past 5 years [20, 21], reflecting a trend similar to that observed in northern Italy and Hungary [16, 26, 36]. Unlike in Italy and Croatia, where *Leishmania* parasites and phleboviruses have been detected in local sand fly populations [3, 4, 10], no such evidence existed for Slovenia prior to this study.

Across the Mediterranean basin, *L. infantum* is commonly found in sand fly populations, with several *Phlebotomus* species confirmed as competent vectors. In France, multiple *Leishmania* strains have been identified in field-collected sand flies [15], while in Turkey and Cyprus, *L. infantum* has been isolated from *Ph. tobbi*, demonstrating the importance of this vector in the eastern Mediterranean [14, 35]. In Croatia, high *L. infantum* seroprevalence in dogs and children suggests a broader distribution of the parasite than previously recognized [33, 42]. However, the main vector species in many parts of the Balkans remains unidentified due to limited entomological data. In Serbia, *L. infantum* DNA was first detected in *Ph. papatasi* in 2017 [39]. In Albania, *Ph. neglectus* has been implicated as the principal vector, supported by parasite isolation and high infection rates in endemic areas [41]. Similarly, in Kosovo, *L. tropica* DNA was detected in *P. neglectus*, suggesting a broader vector potential [19, 40, 44].

Given these findings in neighboring countries, the detection of *Leishmania* DNA in Slovenian sand flies, particularly *Ph. neglectus*, is unsurprising. However, genotyping was unsuccessful due to the low quantity of parasite DNA. This limitation stems from differences in molecular target sensitivity: kinetoplast DNA (kDNA) is present in high copy numbers (10,000–26,000 per parasite), allowing for highly sensitive detection, while the internal transcribed spacer 1 (ITS-1) region, used for species-level identification, exists in far fewer copies. In our experience, ITS-1 nPCR generally yields positive results only when kDNA qPCR Ct values are below 25–30. In this study, the higher Ct values (~36) reflect minimal template availability, likely explaining the failure of ITS-1 amplification despite rigorous PCR conditions, including repeated rounds.

Epidemiologically, the detection of *Leishmania* DNA in *Ph. mascittii* is particularly significant. Although its vector competence has not been experimentally confirmed, mainly due to difficulties in establishing laboratory colonies, its suspected role in *Leishmania* transmission in central Europe has been discussed in previous literature. The presence of *Leishmania* DNA in this species in Slovenia strengthens the hypothesis of its potential involvement in transmission, at least as a permissive host.

The comparative analysis of the two study sites, Cetore (Mediterranean climate) and Velike Žablje (inland Karst climate), revealed differences in seasonal sand fly abundance. In Cetore, sand fly emergence occurred earlier, with a peak in July, while abundance in Velike Žablje peaked in August and extended into the autumn. No sand flies were captured in Velike Žablje in May, suggesting that seasonal activity likely begins there in June. These temporal differences are likely influenced by local climatic factors, including altitude, temperature, and exposure to wind. Such microclimatic variations can significantly affect the phenology, density, and possibly the vectorial capacity of sand fly populations, even within short geographic distances.

## Conclusions

This study provides the first molecular evidence of *Leishmania* DNA in phlebotomine sand flies collected in Slovenia, marking a significant step toward understanding the potential for local transmission of leishmaniasis in the region. Although species-level identification of the parasite was not achieved, the detection of *Leishmania* DNA in both *Phlebotomus neglectus*, a known vector in southeastern Europe, and *P. mascittii*, a species of uncertain but suspected vector competence, highlights the need for continued entomological and epidemiological investigations.

The findings suggest the presence of enzootic *Leishmania* circulation and underscore the potential risk of autochthonous transmission, particularly in areas with established sand fly populations and documented cases of canine leishmaniasis. However, the low parasite DNA loads and absence of species confirmation necessitate cautious interpretation. Further studies should aim to isolate and genotype the parasites, assess vector competence experimentally, and evaluate infection dynamics in local reservoir hosts.

Overall, this work emphasizes the importance of sustained molecular surveillance, improved molecular diagnostic tools, and region-specific vector ecology studies to better assess leishmaniasis risk in Slovenia and Central Europe.

## Supplementary Information

Supplementary Material 1. Figure A1. RT-PCR amplification. Ct 16: Positive control (reared sand fly + Leishmania infantum DNA); Ct 35. Sample 1; Ct: >40: negative control (reared sand fly).

Supplementary Material 2. Figure A2. kDNA RealTime-PCR amplification. Ct 30: Positive control (reared sand fly + *Leishmania infantum* DNA); Ct 35. Sample 2; Ct: >40: negative control (reared sand fly).

Supplementary Material 3. Figure A3. kDNA RealTime-PCR amplification. Ct 30: Positive control (reared sand fly + leishmania DNA); Ct 37. Sample 3; Ct: >40: negative control (reared sand fly).

## Data Availability

Data supporting the main conclusions of this study are included in the manuscript.

## Abbreviation

CDC: Centers for Disease Control and Prevention
