# Supplementary figures and images for: First molecular evidence of Leishmania parasites in sand flies (Diptera: Phlebotominae) from Slovenia
Source: Parasit Vectors. 2025 Aug 22;18:359. doi: 10.1186/s13071-025-07006-4 (PMC12374328; doi:10.1186/s13071-025-07006-4)

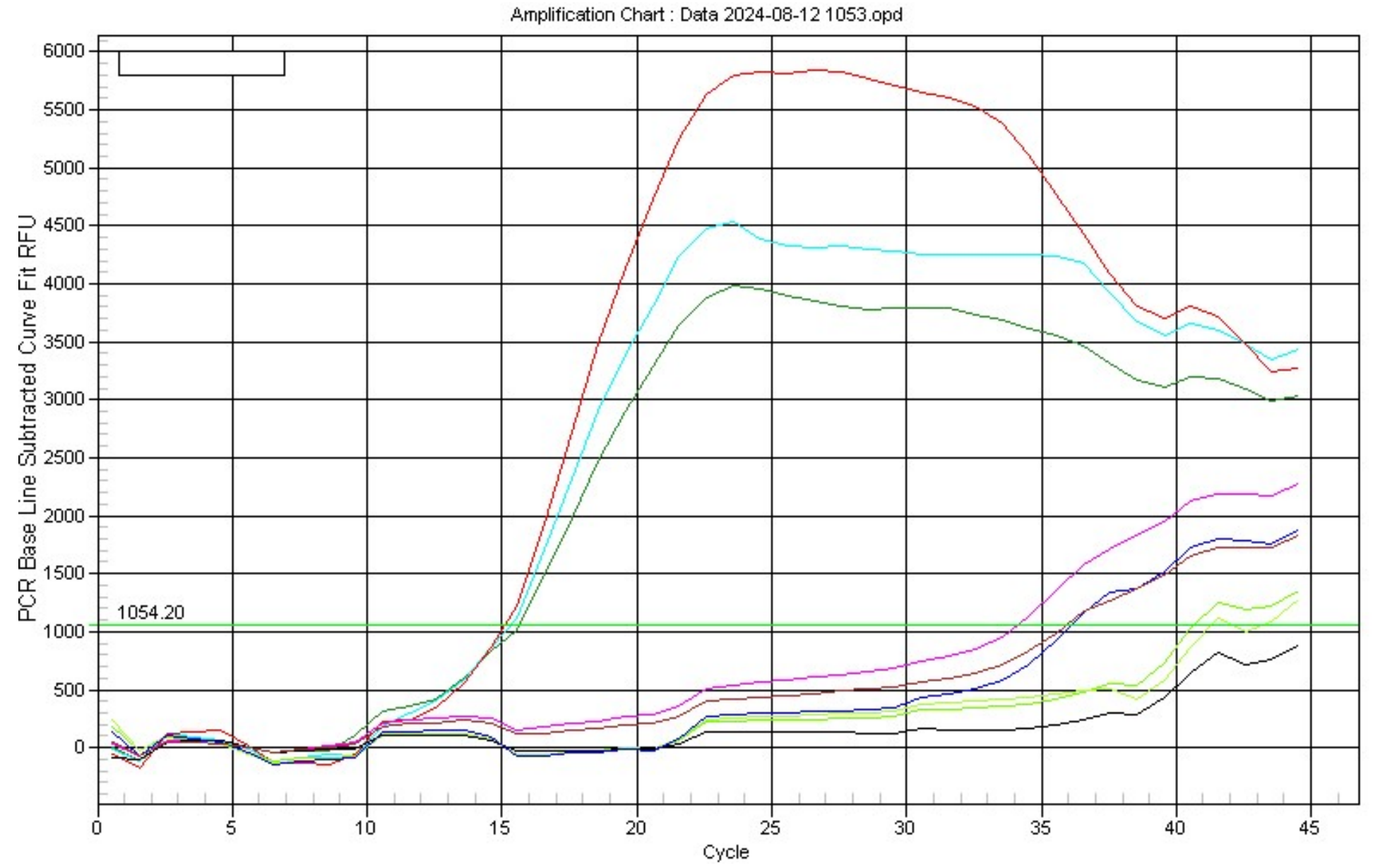

Supplement: Supplementary file 1 — Supplementary Material 1. Figure A1. RT-PCR amplification. Ct 16: Positive control (reared sand fly + Leishmania infantum DNA); Ct 35. Sample 1; Ct: >40: negative control (reared sand fly). [file 13071_2025_7006_MOESM1_ESM.png]

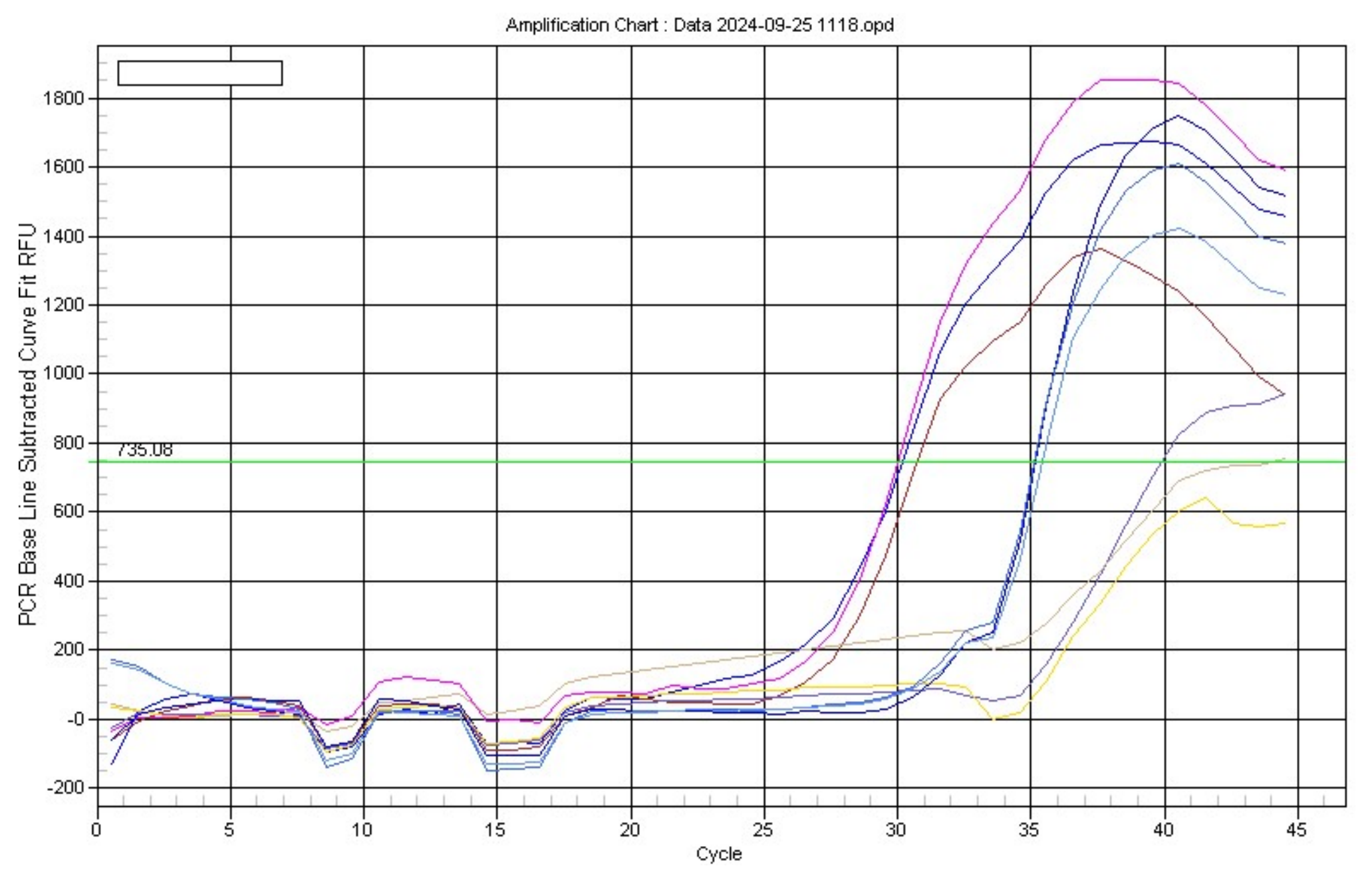

Supplement: Supplementary file 2 — Supplementary Material 2. Figure A2. kDNA RealTime-PCR amplification. Ct 30: Positive control (reared sand fly + Leishmania infantum DNA); Ct 35. Sample 2; Ct: >40: negative control (reared sand fly). [file 13071_2025_7006_MOESM2_ESM.png]

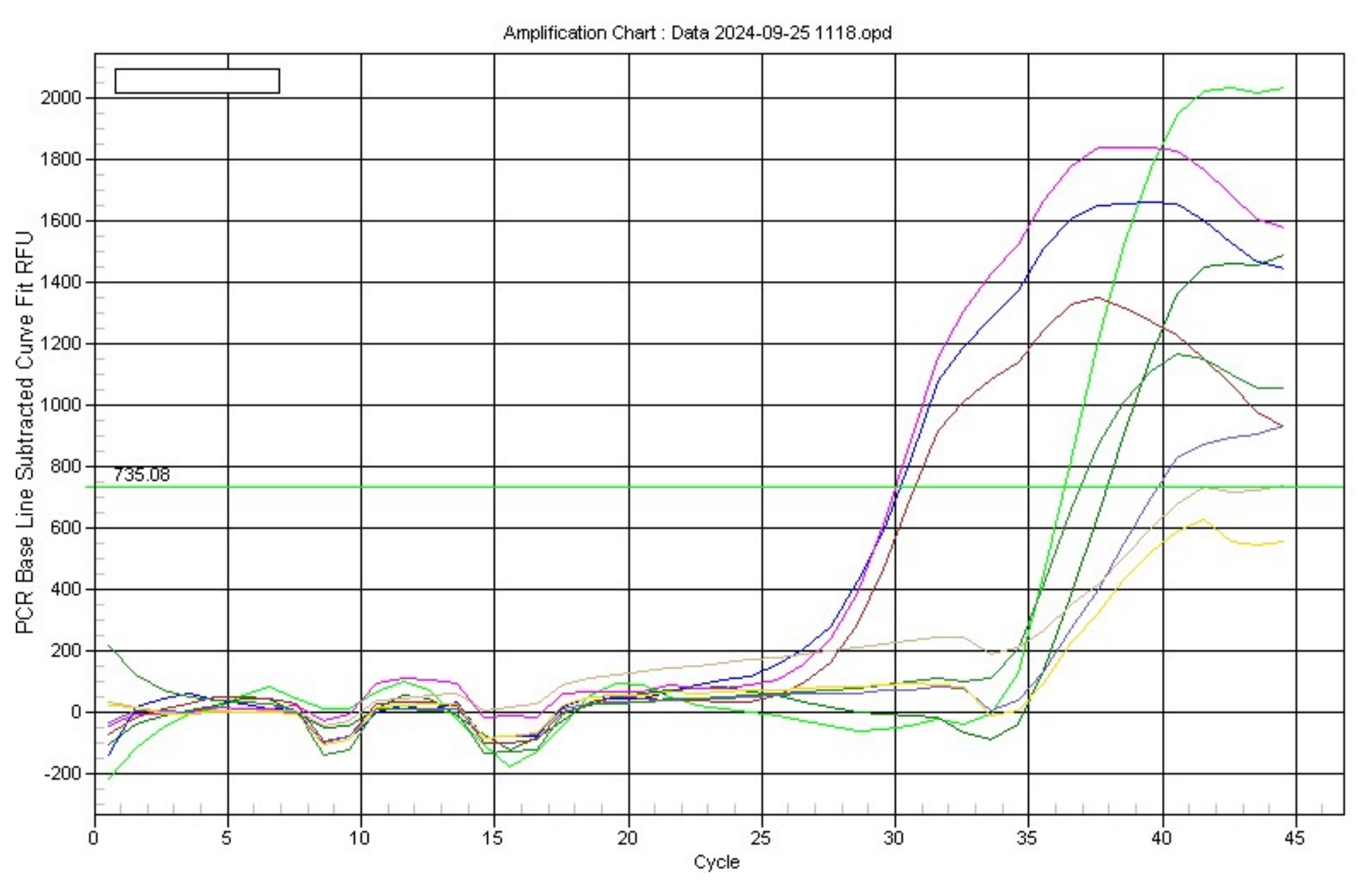

Supplement: Supplementary file 3 — Supplementary Material 3. Figure A3. kDNA RealTime-PCR amplification. Ct 30: Positive control (reared sand fly + leishmania DNA); Ct 37. Sample 3; Ct: >40: negative control (reared sand fly). [file 13071_2025_7006_MOESM3_ESM.png]
